# Bile Acids and FXR: Novel Targets for Liver Diseases

**DOI:** 10.3389/fmed.2020.00544

**Published:** 2020-09-11

**Authors:** Mary Stofan, Grace L. Guo

**Affiliations:** ^1^Department of Pharmacology and Toxicology, Ernest Mario School of Pharmacy, Rutgers University, Piscataway, NJ, United States; ^2^Environmental and Occupational Health Sciences Institute, Rutgers, The State University of New Jersey, Piscataway, NJ, United States; ^3^VA New Jersey Health Care System, Veterans Administration Medical Center, East Orange, NJ, United States

**Keywords:** bile acids, FGF15/19, FXR, agonist, non-alcoholic fatty liver disease, species difference

## Abstract

Bile acids (BAs) are evolutionally conserved molecules synthesized in the liver from cholesterol and have been shown to be essential for lipid homeostasis. BAs regulate a variety of metabolic functions *via* modulating nuclear and membrane receptors. Farnesoid X receptor (FXR) is the most important nuclear receptor for maintaining BA homeostasis. FXR plays a tissue-specific role in suppressing BA synthesis and promoting BA enterohepatic circulation. Disruption of FXR in mice have been implicated in liver diseases commonly occurring in humans, including cholestasis, non-alcoholic fatty liver diseases, and hepatocellular carcinoma. Strategically targeting FXR activity has been rapidly used to develop novel therapies for the prevention and/or treatment of cholestasis and non-alcoholic steatohepatitis. This review provides an updated literature review on BA homeostasis and FXR modulator development.

## Introduction

Bile acids (BAs) serve critical physiological functions, including elimination of cholesterol, absorption of fat and fat-soluble vitamins, regulation of the gut microbiome, and serving as important signaling molecules. BAs are endogenous ligands of farnesoid X receptor (FXR), Takeda G protein receptor 5 *(*TGR5), and sphingosine-1-phosphate receptor 2 (S1PR2). In the liver and intestine, BAs suppress their own synthesis, regulate glucose and lipid homeostasis, and inhibit inflammation and fibrogenesis. Disruption of BA homeostasis leads to severe pathological outcomes, including cholestasis, hepatic steatosis, fibrosis, and liver tumors. Regulating BA pathways has become a novel strategy to treat cholestasis and non-alcoholic steatohepatitis (NASH).

## Overview of BAs

### Synthesis

BAs are amphipathic molecules synthesized from cholesterol in the liver mainly through two pathways, the classical and the alternative pathway (1). In the classical pathway, the initial and rate-limiting step is the 7α-hydroxylation of cholesterol by a cytochrome P450 enzyme, cholesterol 7α-hydroxylase (CYP7A1) (2, 3). The crucial role of CYP7A1 has been demonstrated with *Cyp7a1* knockout (KO) mice that have a high incidence of postnatal death due to abnormal neurological development following vitamin deficiencies (4). Afterwards, microsomal 3β-hydroxy-Δ^5^-C27-steroid dehydroxylase (3β-HSD) converts 7α-hydroxycholesterol to 7α-hydroxy-4-cholestene-3-one (C4) (5), which can be converted by sterol 12α-hydroxylase (CYP8B1) to cholic acid (CA) or alternatively catabolized by cytosolic Δ^4^-3-oxosteroid 5β-reductase (AKR1D1) and 3α-hydroxysteroid dehydrogenase (AKR1C4), yielding a sterol intermediate, 5β-cholestan-3α,7α-diol, which is further converted to chenodeoxycholic acid (CDCA) (5, 6). *Cyp8b1* KO mice eliminated CA synthesis, suggesting that CYP8B1 is required for CA synthesis and is responsible for the CA-to-CDCA ratio in the classical pathway (7). Additionally, the C4 intermediate can be used as a serum marker for assessing BA synthesis levels *in vivo* (8).

In the alternative or acidic pathway, cholesterol is oxidized by mitochondrial sterol 27-hydroxylase (CYP27A1) to produce 27-hydroxycholesterol and 3β-hydroxy-5-cholestenoic acid, which is further hydroxylated by oxysterol 7α-hydroxylase (CYP7B1) to form the intermediate 3β, 7α-dihydroxy-5-cholestenoic acid (6, 9). Subsequent enzymatic conversions produce CDCA.

**Figure 1:**
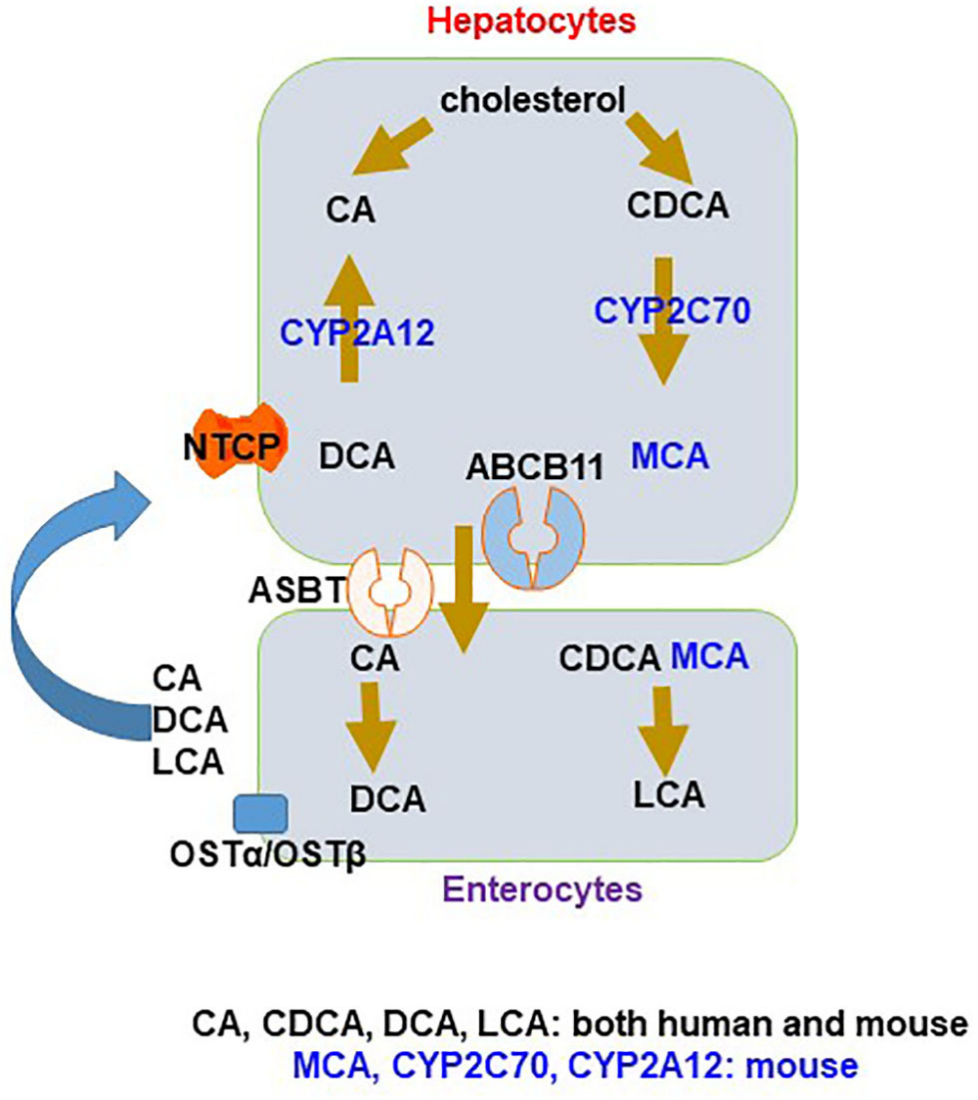
Species difference in bile acid (BA) synthesis and composition. In hepatocytes, primary BAs, cholic acid (CA) and chenodeoxycholic acid (CDCA), are made from cholesterol. In mice, CDCA is converted to muricholic acid (MCA) by CYP2C70. CA and CDCA are conjugated and then efflux *via* ABCB11 to the intestine through the uptake transporter ASBT, where they facilitate lipid absorption. Most BAs are transported back by effluxion out of enterocytes *via* the organic solute transporter (OST)α/OSTβ complex to the liver through portal circulation and taken up into hepatocytes mainly *via* sodium taurocholate co-transporting polypeptide (NTCP), with a small amount being converted to deoxycholic acid (DCA) and lithocholic acid (LCA), the secondary BAs in the large intestine. In mice, DCA can be transported back to the liver and converted to CA by CYP2A12.

There is clear species difference of the composition of BAs between humans and mice (Figure 1). Human primary BAs are CA and CDCA, that form a relatively hydrophobic BA pool consisting of 40% CA, 40% CDCA, and 20% deoxycholic acid (DCA) (9). Mouse primary BAs are CA and muricholic acid (MCA) that is from 6-hydroxylation of CDCA. Hydroxylation significantly changes the physicochemical properties of BAs, resulting in a BA pool that is more hydrophilic, less potent as detergents, and cytotoxic. More significantly, this additional conversion in mice markedly changes BA signaling properties, converting the most potent endogenous FXR agonist (CDCA) to antagonists (MCAs) (9). Three seminal studies discovered the mouse 6β-hydroxylase, CYP2C70, converting CDCA to MCA (10–12). Furthermore, the DCA levels are much higher in humans than in mice because humans are unable to rehydroxylate DCA and lithocholic acid (LCA) whereas mice can. A study by Honda et al. reported that mouse CYP2A12 is the enzyme responsible for 7α-rehydroxylation of taurodeoxycholic acid (TDCA) and taurolithocholic acid (TLCA), solving another unknown of the species difference between humans and mice (12).

Conjugation is considered to be the terminal step in BA synthesis and involves the addition of an amino acid, glycine or taurine, through an amide linkage at carbon 24 (13). Humans and rodents both utilize the enzyme bile acid-CoA:amino acid *N*-acyltransferase (BAAT) for conjugation; however, primary human BAs are mainly conjugated with glycine and, to a less extent, taurine, while rodent primary BAs are taurine conjugates (14, 15). Conjugation of BAs ultimately increases their solubility and amphipathicity (13).

### Enterohepatic Circulation

BAs undergo constant enterohepatic circulation. Conjugated BAs are transported across the canalicular membrane into the bile and stored in the gallbladder in both humans and mice (9). Cholecystokinin, a hormone, is secreted by the duodenum following a meal to stimulate gallbladder contraction, leading to the release of BAs into the intestine (9), where BAs help absorb dietary lipids and fat-soluble vitamins. In the ileum, about 95% BAs are reabsorbed and transported back to the liver through portal circulation (9). Daily, ~0.5 g of BAs, or 5% of the total BA pool, is excreted in the feces, with BAs being recycled 4–12 times a day; this entire process comprises the enterohepatic circulation of BAs (9).

BA transporters are responsible for dynamically moving BAs during the enterohepatic circulation. Efflux of BAs from the hepatocytes into canaliculi is mainly mediated by the bile salt export pump (BSEP; *ABCB11/Abcb11*) (16). The multidrug resistance-associated protein (MRP2; *ABCC2/Abcc2*) effluxes divalent BAs along with other organic substrates, bilirubin conjugates, glutathione, and drugs (17). Like BSEP, MRP2 is an ATP-binding cassette transporter localized to the canalicular membrane of hepatocytes (17). There seems to be a species difference between humans and mice regarding the roles of BSEP and MRP2. Mice use mainly BSEP and, to a smaller extent, MRP2, to efflux BAs into the bile, whereas humans mainly rely on BSEP to efflux BAs into the bile, which could at least partially explain the more severe cholestasis development in human patients with BSEP mutation compared to mice with BSEP deficiency (18). Mutation of the *ABCB11* gene causes BSEP deficiency and progressive familial intrahepatic cholestasis type 2 (PFIC2) (19). PFIC2 is an inherited disorder characterized by severe cholestasis beginning at infancy that can progress to cirrhosis, hepatic failure, hepatocellular carcinoma (HCC), and death (20, 21). Due to the species differences mentioned above, the PFIC2 phenotype cannot be achieved in *Abcb11* KO mice. This leaves a void for a translational model for PFIC2 to study potential therapies as the standard treatment remains to be liver transplantation.

Reabsorption of BAs in the terminal ileum mainly occurs through the uptake mediated by the apical sodium-dependent bile salt transporter (ASBT; *SLC10A2/Slc10a2*) (22), intracellular binding to intestinal bile acid-binding protein (IBABP) (23), and basolateral BA efflux into the portal circulation by the organic solute transporters OSTα and OSTβ heterodimer (24).

At the basolateral (sinusoidal) membrane of hepatocytes, the major BA uptake transporter is the sodium taurocholate co-transporting polypeptide (NTCP; *SLC10A1/Slc10a1*) (25). Interestingly, human NTCP seems to have higher affinity than does the rat transporter, allowing more efficient BA extraction at low plasma levels (25). Sodium-independent basolateral BA uptake into hepatocytes is mediated by organic anion transporting polypeptides (OATPs) (16). Only 25% of the hepatic BA uptake is estimated to be mediated by Na^+^-independent mechanism and responsible for mainly unconjugated BA uptake (16).

Although not directly involved in enterohepatic circulation, an important canalicular membrane flippase encoded by the multidrug resistance gene (*MDR3;ABCB4* in humans and *Mdr2;Abcb4* in mice) is responsible for phospholipid secretion into the bile (26, 27). Disruption of *Mdr2* prevents the secretion of phospholipids, a component of BA mixed micelles, thus increasing the concentration of free BAs that can damage the biliary epithelium (21). Defects in ABCB4 are associated with progressive familial intrahepatic cholestasis type 3 (PFIC3), intrahepatic cholestasis of pregnancy, and adult biliary cirrhosis (28, 29). *Mdr2* KO mice develop severe biliary fibrosis and are a well-established model for primary sclerosing cholangitis (PSC) (30, 31).

The gut microbiota play an important role in BA biotransformation and are responsible for secondary BA formation. Conjugated BAs that remain in the intestine are deconjugated by bacterial bile salt hydrolases (BSHs) (32). In the large intestine, bacterial 7α-dehydroxylase converts CA to DCA and CDCA to LCA through the removal of the hydroxyl group at the C-7 position (32). These secondary BAs are more cytotoxic. While LCA is highly insoluble and mostly excreted by fecal excretion, DCA can be reabsorbed through passive diffusion (33). As mentioned above, mouse hepatocytes can rehydroxylate DCA to CA by CYP2A12 (12). Species differences in the gut microbiota may affect the generation of secondary BAs and should be considered when using animal models to study human BA signaling (21).

BAs are important for the intestinal absorption of lipids and lipid-soluble nutrients, removal of excess cholesterol, regulating bile flow, modulating the gut microbiome, and modulating energy homeostasis. Many of these functions are performed by modulating a nuclear receptor (NR) FXR in a tissue-specific manner. Additional NRs and membrane-bound receptors that have been identified to be activated by BAs include pregnane X receptor (PXR), vitamin D receptor (VDR), Takeda G protein-coupled receptor (TGR5), and sphingosine-1-phosphate receptor 2 (S1PR2) (34–36).

## Farnesoid X Receptor

FXR is the most important NR to regulate BA homeostasis. NRs are ligand-activated transcription factors that regulate the expression of genes involved in various processes, including cell growth, differentiation, and metabolism (37). The general structure of NRs consists of an N-terminal DNA-binding domain (DBD) and a C-terminal ligand-binding domain (LBD), with the DBD being the most conserved area that contains two zinc finger motifs (9). These zinc fingers allow the NR to bind to DNA elements, known as hormone response elements (HREs), composed of direct, inverted, or everted repeats of the sequence AGGTCA and separated by a variable number of nucleotides (38). NR activation also requires either homodimerization or heterodimerization with retinoid X receptor (RXR) (39).

FXR was originally labeled as an orphan NR (38). After multiple groups demonstrated that physiological concentrations of free or conjugated BAs could activate FXR, with CDCA being the most potent, followed by DCA, CA, and LCA, BAs were recognized to be the endogenous ligands of FXR and FXR is now considered an “adopted” NR (40–42). FXR is highly expressed in the liver, ileum, kidneys, and adrenal glands (40). The most common FXR response element (FXRE) consists of an inverted AGGTCA repeat separated by one nucleotide (IR1); FXR could also bind to an everted repeat separated by two nucleotides (ER2) (43). Both steroidal and non-steroidal FXR agonists are being developed in the treatment of various liver diseases and include semi-synthetic BA obeticholic acid (OCA), cilofexor, and tropifexor, with OCA being used clinically to treat primary biliary cholangitis (PBC).

### Regulation of BA Homeostasis

**Figure 2:**
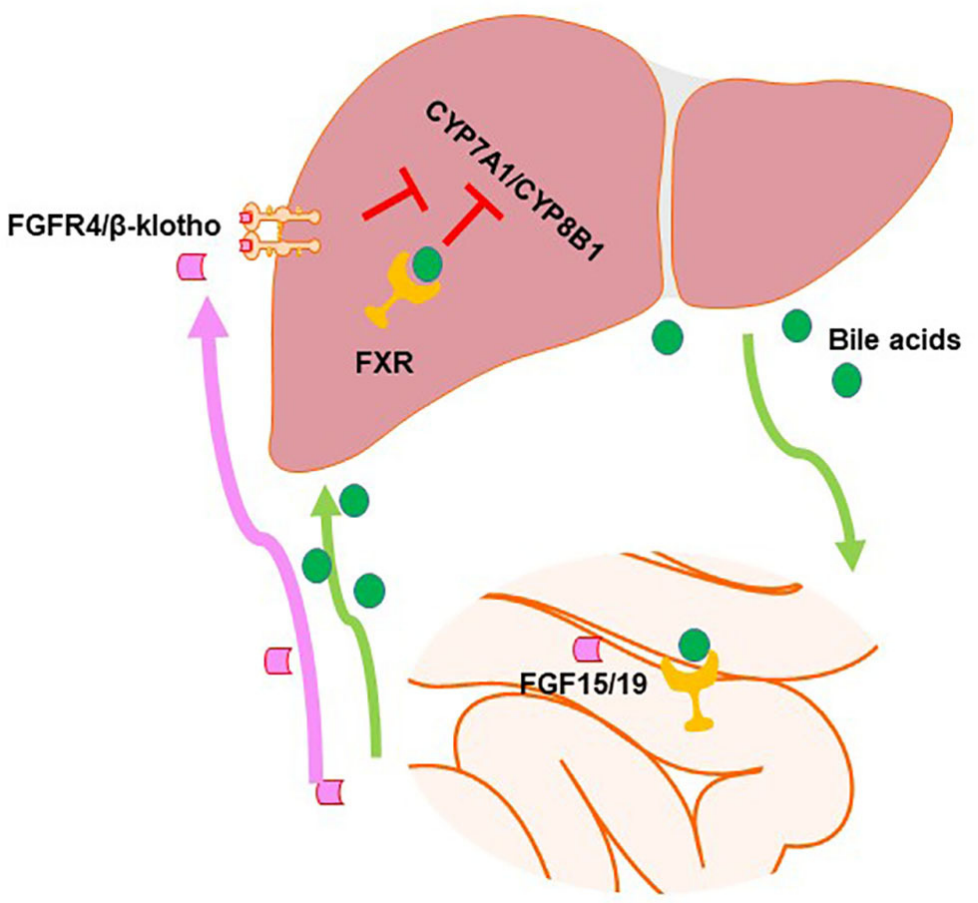
Farnesoid X receptor (FXR) regulates bile acid (BA) synthesis in a tissue-specific manner. In the intestine, FXR activation induces fibroblast growth factor (FGF)15/19, which can go to the liver and activate the FGFR4/β-klotho dimer to activate signaling pathways in order to inhibit the expression of genes in the classical BA synthesis pathway. Hepatic FXR activation also inhibits BA synthesis, albeit to a smaller degree.

It has been well-established that FXR is involved in the regulation of BA homeostasis. As shown in Figure 2, there is a clear tissue-specific role of FXR in the liver and intestine to regulate BA synthesis (44). Activation of intestinal FXR plays a major role and activation of liver FXR serves a minor role in suppressing *CYP7A1/Cyp7a1* gene expression through the induction of the ileal hormone fibroblast growth factor 19 (FGF19) in humans and FGF15 in mice and hepatic small heterodimer partner 1 (SHP-1), respectively (42, 44–47). In contrast, *Cyp8b1* gene repression *via* FXR is almost equally dependent on both intestinal and liver FXR (44). Furthermore, FXR is critical in regulating the enterohepatic circulation of BAs by inducing the expression of BSEP, IBABP, and OSTα/β and suppressing those of NTCP and ASBT (48–52).

### Regulation of Lipid and Glucose Homeostasis

FXR also shows critical effects in regulating lipid and glucose homeostasis. In general, FXR activation leads to lower lipid levels in the circulation as it suppresses *de novo* fatty acid synthesis (53, 54), decreases very low-density lipoprotein (VLDL) hepatic secretion (55), and increases triglyceride hydrolysis and clearance as well as fatty acid oxidation (56–60). Activation of FXR may reduce glucose intolerance by reducing hepatic gluconeogenesis and glycolysis and increasing glycogen synthesis (61). FXR activation may decrease gluconeogenesis *via* SHP-mediated suppression of the critical transcription factors involved in gluconeogenesis (62). In contrast, a different study utilizing human and rat hepatocytes and mouse livers showed that FXR agonism induced phosphoenolpyruvate carboxykinase (PEPCK) expression and glucose levels (63). Our genome-wide ChIP-seq analysis also suggests that FXR could regulate glucose homeostasis, but there may be species differences among humans and mice (43, 64). Despite conflicting evidence, it is apparent that FXR may play important roles in glucose homeostasis as FXR KO mice develop fatty livers, elevate circulating free fatty acids (FFAs) and serum glucose levels, and present insulin resistance (65). In both diabetic db/db and wild-type mice, FXR activation or hepatic overexpression significantly lowered the blood glucose levels, decreased the FFA levels, and increased the insulin sensitivity (66), suggesting FXR activation may improve metabolic syndrome.

### Role in Inflammation and Fibrosis

During liver injury, FXR has been shown to play an anti-inflammatory role (67, 68). Monocyte chemoattractant protein-1 (MCP-1/CCL2) is a key chemokine that regulates the migration and infiltration of monocytes/macrophages (69). In the methionine/choline-deficient (MCD) diet-induced NASH model, the synthetic FXR agonist WAY-362450 decreased MCP-1 expression and significantly decreased inflammatory cell infiltration in the liver (68). Nuclear factor kappa-light-chain enhancer of activated B cell (NF-κB) is a transcription factor that induces the expression of various pro-inflammatory genes (70). FXR KO mice displayed strong hepatic inflammation after treatment with lipopolysaccharide (LPS), confirmed by massive liver necrosis and the significant increase in the hepatic cytokine signaling molecules inducible nitric oxide synthase (iNOS), cyclooxygenase-2 (COX-2), and interferon-γ (IFN-γ) (67). Ultimately, the pretreatment of HepG2 cells and mouse primary hepatocytes with FXR agonists suppressed the NF-κB-mediated inflammation in an FXR-dependent manner (67). FXR could suppress inflammation *via* an indirect mechanism by reducing cholestasis and the levels of toxic BA production and accumulation in the liver, as described above.

FXR activation suppresses the development of hepatic fibrosis. In addition to regulating hepatic lipid metabolism and reducing hepatic fibrosis, FXR seems to directly inactivate hepatic fibrosis by inducing anti-fibrotic gene expression in hepatic stellate cells (HSCs). Activation of FXR induces SHP to increase the peroxisomal proliferator-activated receptor γ (PPARγ) expression in HSCs, and PPARγ is well-known to inactivate HSCs (71, 72). Recently, we have shown that FGF15 deficiency reduces hepatic fibrosis through increasing FXR activation following loss of FGF15-mediated suppression of BA synthesis (73, 74). Interestingly, in a human HSC cell line, LX2, FGF19 does not suppress fibrogenic gene expression, but suppresses inflammation, likely through modulating the inhibitor of nuclear factor kappa B (IκB) activity (74). These studies provide another group of evidence to support the role of FXR as a homeostatic regulator to suppress liver inflammation and fibrosis.

### Role in Cholestasis

There is conflicting evidence regarding the role of FXR in cholestatic diseases. In an early study, the synthetic FXR agonist GW4064 was investigated in rat models of extrahepatic and intrahepatic cholestasis through bile duct ligation (BDL) and α-naphthylisothiocyanate (ANIT) administration, respectively (75). Significant reductions in liver injury were observed in GW4064-treated animals in both cholestatic models, revealed by the reduced alanine aminotransferase (ALT) and aspartate transaminase (AST), necrosis, inflammation, and bile duct proliferation (75). The observed protective effects of GW4064 suggest that FXR agonists may be helpful in treating cholestatic diseases (75).

However, another group found that FXR KO mice were protected from obstructive cholestasis achieved through BDL (76). In FXR KO mice after BDL, mortality and liver injury were reduced as serum bilirubin was not significantly elevated (76). FXR KO mice had reduced serum total BA concentrations and had a marked induction of the basolateral transporter multidrug resistance-associated protein 4 (*Mrp4*), suggesting that these animals had a greater capacity to export BAs back into circulation and reduce hepatoxicity (76). This study supports the potential clinical use of FXR antagonists in the treatment of obstructive cholestatic diseases (76).

Looking further into the role of FXR during intrahepatic cholestasis, ANIT-induced injury was utilized in wild-type (WT), FXR KO, and PXR KO mice (77). Serum ALT, alkaline phosphatase (ALP), and bilirubin were elevated in all genotypes after ANIT administration, with the highest ALP levels seen in FXR KO mice (77). ANIT-treated FXR KO mice had higher concentrations of serum and liver unconjugated BAs across all genotypes (77). While ANIT treatment induced the messenger RNA (mRNA) expressions of *Mdr2, Bsep*, and ATPase, class I, type 8B and member 1 *(Atp8b1)* in WT and PXR KO mice, no upregulation was observed in FXR KOs (77). It was concluded that FXR deficiency, not PXR deficiency, was responsible for the increased susceptibility to injury in the ANIT-induced intrahepatic cholestasis model due to the reduction of hepatobiliary efflux transporters and the accumulation of unconjugated BAs (77). Furthermore, pretreatment of the FXR agonist GW4064 was also investigated in ANIT-treated WT mice (77). GW4064 treatment was shown to be protective as it reduced necrosis compared to ANIT treatment alone (77). This reproduces what Liu et al. had found in BDL and ANIT-induced injury in rats and further supports FXR as a therapeutic target for intrahepatic cholestasis (77).

Through the use of reversible BDL (rBDL) in the rat to model cholestasis, FXR activation by OCA worsened the biliary injury, shown by a considerable increase in ALT and ALP compared to the controls (78). OCA treatment in rBDL rats upregulated *Bsep*, multidrug resistance-associated protein 3 (*Mrp3*), *Mrp4*, and Ostβ transporters (78). The 8-fold induction of the FXR target gene *Bsep* was suggested to be the cause of biliary injury as BAs would be pumped *via* BSEP into an already obstructed biliary tree (78).

### Cholangiocytes

Cholangiocytes are epithelial cells which line the bile ducts of the biliary tree (79). Through absorptive and secretory transport systems in cholangiocytes, bile is modified to become more fluid and alkaline (80). Bile then enters the gallbladder for concentration and storage or delivered to the intestinal lumen (80). Cholangiocytes have also been shown to be actively involved in bile homeostasis (81). Compared to hepatocytes, cholangiocytes have no or low expressions of *Cyp7a1* and *Cyp8b1*, but considerable expression of *Cyp27a1*, suggesting that cholangiocytes are involved in cholesterol metabolism (81). Measurement of the mRNA levels revealed that Fgf15 was expressed at higher levels in cholangiocytes compared to hepatocytes, while the fibroblast growth factor receptor 4 (Fgfr4) expression was lower (81). As FXR is known to regulate Fgf15/FGF19 levels, investigation of a similar regulation in cholangiocytes was achieved through treatment of rat cholangiocytes with CDCA and the FXR agonist GW4064, with both treatments inducing the expression of Fgf15 (81). Additionally, cultured human cholangiocytes treated with CDCA induced the secretion of FGF19 in the medium (81). FGF15/19-mediated repression of *Cyp27a1* in cholangiocytes was found to differ from hepatocytes and is mediated through p38 kinase (81). Ultimately, understanding BA metabolism in cholangiocytes may help provide therapeutic pathways for cholangiopathy treatments (81).

One of the most common biliary complications after liver transplantation (LT) is non-anastomotic strictures that develop after biliary epithelial damage and can result from BA toxicity (82). To investigate the mechanism of cholangiocyte BA transport following LT, a rat LT model was utilized. After transplantation, a prolonged biliary transport time of BAs was observed, while the expression of FXR was dramatically decreased and was related to cold ischemic time of the donor liver. Furthermore, *in vitro*-cultured human biliary epithelial cells under hypoxic conditions exhibited a repression of FXR expression and DNA binding activities (82). Hypoxic conditions also altered the expressions of BA transporters as hypoxia slightly induced Asbt expression and repressed both Ostα and Ostβ (82). This led to the intracellular accumulation of BAs, increased cell apoptosis, and increased expression of profibrotic factors in cholangiocytes (82). It was concluded that, after LT, repression of FXR under ischemic/hypoxic conditions led to the disruption of BA transport of cholangiocytes and, thus, biliary damage (82).

## Fibroblast Growth Factors 15/19

Fibroblast growth factors (FGFs) make up a family of at least 22 proteins that regulate various biological processes including growth, development, and differentiation (83, 84). FGF15 and its human ortholog FGF19 belong to the subfamily of endocrine FGFs that act as hormones due to their low or no affinity for heparin sulfate, which allows them to enter systemic circulation (84, 85). FGF19 was originally identified in the fetal brain during a screen for novel FGFs (86). Although FGF15 and FGF19 are orthologs, they interestingly only share ~50% amino acid identity (86). For high-affinity receptor binding, the endocrine FGFs require klotho proteins that interact with fibroblast growth factor receptors (FGFRs) (87). β-Klotho specifically binds to FGF15/19 which has high affinity for fibroblast growth factor receptor 4 (FGFR4) and less for fibroblast growth factor receptor 1 (FGFR1) that are highly expressed in hepatocytes and white adipose tissue (WAT), respectively (88, 89). Low levels of FGFR4 expression are also detected in other cell types, including HSCs, macrophages, and some central neurons (90). FGF15/19 is expressed in ileal enterocytes, where it is strongly induced by FXR activation (84). Once released into blood circulation, FGF15/19 acts on the liver to repress BA synthesis, as described above.

However, mouse FGFR4 does not recognized human FGF19 (91). Therefore, when using high dosage of FGF19 in mice, the observed effects may be due to the activation of FGFR1 or other FGFRs, but not FGFR4 by FGF19.

### Role in Energy Expenditure

To investigate the role of FGF19 in physiological homeostasis, transgenic mice expressing human *FGF19* were utilized (92). *FGF19* transgenic mice had a significant reduction in fat mass arising from an increase in energy expenditure (92). When fed a high-fat diet, *FGF19* transgenic mice did not become obese or diabetic (92). The results suggest two mechanisms by which FGF19 may increase energy expenditure through an increase in brown adipose tissue (BAT) and through a decrease in liver enzyme acetyl CoA carboxylase 2 (ACC2) (92). Reduction in ACC2, the rate-limiting enzyme for fatty acid entry into the mitochondria, also resulted in reduced liver triglyceride levels (92). In an additional study, FGF19 increased the metabolic rate in mice fed a high-fat diet while reducing body weight and diabetes in leptin-deficient mice (93). FGF19 also acts in the central nervous system to improve insulin sensitivity by reducing hypothalamic agouti-related peptide (AGRP)/neuropeptide Y (NPY) neuron activity (94). In summary, FGF15/19 increases insulin sensitivity, thermogenesis, and weight loss and decreases serum cholesterol and triglyceride levels.

### Protein and Glycogen Synthesis

FGF15/19 also regulates hepatic protein and glycogen synthesis (95). *Fgf15* KO mice were shown to be glucose-intolerant and store half as much hepatic glycogen compared to control wild-type mice (95). In diabetic mice lacking insulin, FGF19 treatment restored the hepatic glycogen concentrations to normal levels, indicating that FGF19 activates an insulin-independent pathway to regulate glycogen metabolism (95). It was determined that FGF15/19 uses a RAS/extracellular signal-regulated protein kinase (ERK)/p90RSK pathway to induce hepatic glycogen and protein synthesis *in vivo* (95). FGF19 also shows a positive effect on muscle weight, revealed by a study showing that FGF19 stimulates the phosphorylation of the ERK1/2 and the ribosomal protein S6 kinase (S6K1), an mTOR-dependent master regulator of muscle cell growth (96).

### Gluconeogenesis

Energy homeostasis is additionally regulated through FGF15/19 repressing gluconeogenesis, like insulin (97). While insulin peaks in serum 15 min after feeding, FGF15/19 peaks ~45 min later due to the increase of BAs in the small intestine (97). *In vivo*, FGF15/19 blocks the expression of gluconeogenesis genes through the dephosphorylation and inactivation of the transcription factor cAMP regulatory element-binding protein (CREB) (97). This then inhibits the expression of peroxisome proliferator-activated receptor-γ coactivator-1α (PGC-1α) and other downstream hepatic metabolism genes (97).

### Fatty Acid Synthesis

Lastly, FGF19 inhibits hepatic fatty acid synthesis. Primary hepatocytes incubated with recombinant FGF19 protein in the presence or absence of insulin showed that FGF19 suppressed the insulin-dependent stimulation of fatty acid synthesis (98). Similar to the SHP-mediated suppression of sterol regulatory element-binding protein 1c (SREBP1c) following FXR activation, FGF19 was shown to decrease SREBP1c through increasing the signal transducer and activator of transcription 3 (STAT3) and decreasing the peroxisome proliferator-activated receptor-γ coactivator-1β (PGC-1β), while also increasing the expression of SHP (98). This favorable inhibition of hepatic fatty acid synthesis, along with the promotion of protein and glycogen synthesis and the repression of gluconeogenesis, supports the beneficial effects of FGF15/19 on metabolic syndrome and warrants further investigation of FGF15/19 in the prevention and treatment of NASH. Indeed, modified FGF19 has been shown to be beneficial in mouse models of NASH and cholestasis (99, 100).

## FXR as Drug Targets—FXR Agonists

There are many FXR modulators that have undergone clinical trials for the treatment of chronic liver diseases. The focus for most of these trials is the efficacy of FXR activation on cholestasis, NASH, and obesity; however, there are some studies focused on minor indications, including bile acid diarrhea or association with reactivation of latent pro-virus (clinical trials.gov). Currently, two types of FXR agonists—steroidal represented by OCA *vs*. non-steroidal represented by tropifexor—are front-runners for obtaining U.S. Food and Drug Administration (FDA) approval for the treatment of NASH.

The first FDA-approved FXR agonist for the treatment of PBC is OCA, which is a steroidal FXR agonist modified from CDCA (101). When compared to CDCA, OCA was shown to be ~100 times more potent (101). In a model of cholestasis, male Wistar rats were administered LCA through an intravenous infusion to impair bile flow. Administration of OCA alone did not induce cholestasis, while co-infusion of LCA and OCA fully reversed bile flow impairment and protected hepatocytes from necrosis (101). This initial study confirmed OCA as a selective, potent FXR agonist and warranted further investigation of additional therapeutic uses.

The traditional first-line treatment for PBC is ursodeoxycholic acid (UDCA) as it has been shown to improve liver tests and transplant-free survival with minimal side effects (102). However, not all patients respond to UDCA (102). In a randomized, double-blinded, 12-week, phase II clinical trial, the efficacy of OCA in PBC patients who did not respond favorably to UDCA was evaluated (103). Patients (*n* = 165) were randomly assigned to receive 10, 25, or 50 mg of OCA or placebo once daily in addition to an existing dose of UDCA (103). The primary endpoint was level change of ALP from baseline until the conclusion of the study (103). All three doses significantly reduced the levels of ALP, γ-glutamyltransferase (GGT), and ALT compared to placebo. However, pruritus was reported in all groups, with severity correlating to the dose of OCA (103). Based on the efficacy and tolerability, the once daily dose of 10 mg OCA was determined to be the most effective (103).

In the randomized, double-blinded, phase III POISE trial, 217 PBC patients who had an inadequate response to UDCA were assigned to receive 10 mg OCA, 5–10 mg OCA, or placebo once daily for 12 months (104). Patients still received UCDA as a background therapy (104). The primary endpoint was a reduction in ALP from baseline and a normal total bilirubin level, which was reached in more patients in both OCA groups compared to placebo (104). As seen previously in the phase II trial, an OCA dose-dependent increase in the incidence of pruritus was reported (104). Based on the favorable effects of OCA on important biochemical markers, the FDA approved OCA for the treatment of PBC patients with an inadequate response of intolerance to UDCA in 2016 (105).

Another phase II study investigated OCA as a monotherapy in PBC patients (106). Patients received 10 mg OCA, 50 mg OCA, or placebo once daily for 3 months and then followed up for up to 6 years (106). OCA treatment as a monotherapy significantly improved ALP and other biochemical markers associated with improved clinical outcomes (106). However, severe pruritus was reported in almost all patients who received 50 mg OCA (106). Compared to UDCA co-therapy, no additional benefits for OCA as a monotherapy were reported (107).

A multiyear study (COBALT) to determine the effects of OCA in PBC patients with more advanced liver disease is ongoing (104). In 2017, after 11 cases of serious liver injury and 19 cases of death associated with OCA were reported, the FDA released a black box warning for the use of OCA in patients with decompensated cirrhosis (105). Many of the cases of increased liver injury appeared to be due to inappropriate high dosing of OCA (105).

Due to arising side effects including pruritus and increased risk of liver decompensation in cirrhotic PBC patients administered OCA, a study to determine whether OCA worsened liver injury under cholestatic conditions was carried out (108). BDL and ANIT treatment were studied in rats (108). In both models, OCA treatment exacerbated liver injury in a dose-dependent manner and downregulated the expression of basolateral transporters (108). The non-steroidal FXR agonist GW4064 was also tested in the ANIT cholestasis model. In contrast, GW4064 administration decreased the severity of cholestatic injury compared to OCA and reduced AST, ALT, GGT, and bilirubin (108). This is again consistent with the results published by Liu *et al*. (75) and suggests that the safety of FXR agonists is impacted by their pharmacokinetic properties (108). OCA, as a semi-synthetic derivative of CDCA, has a high rate of intestinal absorption, which allows it to recirculate like endogenous BAs (108). While synthetic GW4064 undergoes taurine conjugation in the liver which is then not recognized by intestinal transporters thus reducing its bioavailability (108). Under cholestatic conditions, OCA accumulates in the liver where it may reach toxic concentrations (108, 109). In mice, genetic KO of FXR or inhibition of FXR both resulted in protection from injury induced by OCA in an ANIT model of cholestasis (108). After RNAseq analysis, FXR antagonism was shown to reverse the transcription of over 2,000 genes, including V-Maf avian musculoaponeurotic fibrosarcoma oncogene homolog G (Mafg) and its partner nuclear factor erythroid 2-related factor 2 (Nrf2) (108). Mafg expression has been shown to be induced in cholestatic diseases and represses genes involved in the synthesis of antioxidant glutathione (110, 111). The modulation of these transcription factors was then investigated. Pharmacologic or genetic inhibition of Mafg prevented damage caused by ANIT and OCA, while Nrf2 induction was protective. These results support that the negative side effects of OCA treatment are FXR-mediated (108).

There is currently no approved treatment for PSC, and the efficacy of UDCA for PSC remains uncertain (112). Thus, the efficacy and safety of OCA in PSC patients were assessed in a phase II randomized, double-blind, placebo-controlled, dose-finding study (113). Patients (*n* = 76) were assigned to receive 1.5–3.0 mg OCA, 5–10 mg OCA, or placebo once daily for 24 weeks (113). At 24 weeks, treatment with 5–10 mg OCA significantly reduced serum ALP compared to placebo (113). Dose-related pruritus was reported as the most common side effect, consistent with the earlier clinical studies (113).

The safety and efficacy of the non-steroidal FXR agonist cilofexor (GS-9674) were evaluated in a phase II double-blinded, placebo-controlled study in PSC patients (114). Randomized patients received 100 mg cilofexor, 30 mg cilofexor, or placebo once daily for 12 weeks (114). Treatment with cilofexor was generally well-tolerated, safe, and improved the biochemical markers of cholestasis and inflammation (114). Significant dose-dependent reductions in serum ALP, GGT, ALT, and AST with cilofexor compared to placebo were reported (114). The effect of cilofexor on ALP was independent of UDCA use, and adverse events were similar between treatment groups (114).

Cilofexor was also evaluated in a double-blind, placebo-controlled, phase II trial in patients with NASH (115). Non-cirrhotic patients (*n* = 140) were randomized to receive 100 mg cilofexor, 50 mg cilofexor, or placebo once daily for 24 weeks (115). Cilofexor was safe and significantly improved hepatic steatosis, liver biochemistry (e.g., GGT), and bile acids (115). Compared to OCA treatment that resulted in increases in serum LDL-C and total cholesterol, cilofexor treatment had no significant effects on serum lipids (115). Moderate to severe pruritus was reported in 14% of the 100-mg cilofexor group and 4% of the 30-mg group (115). In contrast, 23% of the OCA-treated patients reported pruritus (116). However, cilofexor treatment only had modest beneficial effects on liver biochemistry compared to OCA treatment, indication of a potential limitation for efficacy (115).

To evaluate the effect of FXR activation by OCA on insulin resistance and liver lipid metabolism, Zucker (*fa*/*fa*) rats that contain a loss-of-function mutation in the hunger hormone leptin receptor were utilized (117). This mutation leads to hyperphagia and hyperleptinemia, resulting in diabetes, insulin resistance, obesity, and liver steatosis; therefore, Zucker (*fa*/*fa*) rats are considered a non-alcoholic fatty liver disease (NAFLD) model (117). Daily OCA treatment (10 mg/kg) over 7 weeks reversed insulin resistance and prevented body weight gain and liver fat deposition (117). Moreover, OCA treatment reduced blood triglyceride and plasma aminotransferases and improved liver histopathology (117). Reversal of insulin resistance after the administration of OCA is further supported by *in vitro* data showing that OCA significantly increases insulin secretion in mouse β-TC6 cells and human pancreatic islets (118). Additionally, OCA activation of FXR in mouse β-TC6 cells leads to AKT (protein kinase B)-dependent translocation of glucose transporter 2 (GLUT2), thus increasing the glucose uptake by these cells (118). Taken together, OCA activation of FXR improves hyperglycemia through enhanced insulin secretion and glucose uptake by the liver (118).

OCA has also been shown to exhibit anti-inflammatory and anti-fibrotic properties. While investigating the NF-κB signaling pathway, a key inflammation pathway, pretreatment of HepG2 cells with OCA (3 μM) inhibited the expression of the cytokine-inducible enzymes COX-2 and iNOS after stimulation with LPS or tumor necrosis factor alpha (TNFα) (67). Inhibition of iNOS by OCA was also confirmed in LPS-treated primary mouse hepatocytes (67).

After animal studies showed that OCA decreased insulin resistance and hepatic steatosis, the efficacy and safety of OCA were first evaluated in a phase IIa study in patients with type II diabetes and non-alcoholic fatty liver disease (119). The participants were randomly assigned to placebo (*n* = 23), 25 mg OCA (*n* = 20), or 50 mg OCA (*n* = 21) groups for the 6-week treatment period (119). Both OCA groups exhibited reduced GGT and ALT levels along with decreased bodyweight (119). Furthermore, treatment of OCA led to improved insulin sensitivity and elevated FGF19 serum levels. This, in conjunction with the decreased BA precursor C4 and endogenous BAs, again confirmed OCA's FXR agonist activity (119).

Based on previous favorable results, OCA was further investigated in the phase IIb Farnesoid X Receptor Ligand Obeticholic Acid in NASH Treatment (FLINT) trial (116). In this multicenter, double-blind, randomized clinical trial, patients with non-cirrhotic NASH were assigned to receive 25 mg OCA (*n* = 141) daily or placebo (*n* = 142) for 72 weeks (116). OCA treatment was shown to improve the biochemical and histological features of NASH when compared with placebo; specifically, 45% of OCA patients improved their NAFLD activity score by two points or greater without worsening of fibrosis compared to the 21% improvement in placebo patients (116). However, there was no significant difference in the histological resolution of NASH between the OCA-treated and placebo groups (120). Adverse outcomes of pruritus and unfavorable dyslipidemia manifested in the OCA treatment group (116). Additionally, the favorable effects on ALP, lipids, and blood glucose seen in the placebo group associated with weight loss were absent or reversed in the OCA-treated patients (120).

Currently, OCA is being evaluated by Intercept in a phase III trial REGENERATE (121). To assess OCA's effect on liver histology and clinical outcomes, 2,065 biopsy-confirmed NASH patients were randomized into a 10-mg OCA, 25-mg OCA, or placebo group (121). Total study duration is estimated to be 6 years, with interim biopsies performed after the first 18 months to evaluate improvement of fibrosis stage and resolution of NASH with no worsening fibrosis (121). Although OCA was recently approved by the FDA for treating PBC, the current American Association of the Study of Liver Diseases guidelines do not recommend the off-label treatment of OCA in NASH patients until further safety and efficacy data are available (122). In February 2019, Intercept announced that OCA achieved the primary endpoint of improving liver fibrosis without worsening of NASH after 18 months (*p* = 0.0002). This marks the first and largest successful phase 3 study in fibrosis patients due to NASH. Intercept filed a New Drug Application (NDA) with the FDA in September 2019. As of June 2020, the FDA issued a complete response letter stating that the predicted benefit of OCA did not outweigh the potential risks in patients with fibrosis due to NASH and that long-term outcome needs to be evaluated (123). Thus, accelerated approval was not granted at this time.

Tropifexor is a representative of non-steroidal FXR agonists. In mouse models of NASH, tropifexor significantly reduced oxidative stress, steatosis, inflammation, and fibrosis (124). It will be very interesting to see whether, as a non-steroidal FXR agonist, tropifexor will present similar adverse effect to the steroidal FXR agonists.

## Conclusion

As a key regulator of BA homeostasis, FXR activation suppresses BA synthesis mainly through the induction of FGF15/19 in the gut and promotes enterohepatic BA circulation. FXR agonism also regulates lipid metabolism, reduces hepatic gluconeogenesis and glycolysis, and increases glycogen synthesis while playing an anti-inflammatory role during liver injury. FGF15/19 favorably increases energy expenditure and glycogen synthesis while decreasing gluconeogenesis and fatty acid synthesis. While FXR and FGF19 have been considered promising targets for the treatment of cholestasis and NASH, the molecular mechanism by which these two factors regulate liver BA transport, steatosis, and inflammation needs to be further determined, and most importantly, an individualized treatment plan is paramount to develop drugs and treatment strategy with better efficacy and less toxic effects.

## Data Availability Statement

The raw data supporting the conclusions of this article will be made available by the authors, without undue reservation.

## Author Contributions

MS and GG have contributed to the writing and editing of the manuscript. All authors contributed to the article and approved the submitted version.

## Conflict of Interest

The authors declare that the research was conducted in the absence of any commercial or financial relationships that could be construed as a potential conflict of interest.

## Abbreviations

ALP: Alkaline phosphatase
ALT: Alanine aminotransferase
ANIT: α-Naphthylisothiocyanate
ASBT: Apical sodium-dependent bile salt transporter
AST: Aspartate transaminase
BA: Bile acid
BDL: Bile duct ligation
BSEP: Bile salt export pump
C4: 7α-Hydroxy-4-cholesten-3-one
CA: Cholic acid
CDCA: Chenodeoxycholic acid
CY27A1: Cytochrome P450 27A1
CYP7A1: Cytochrome P450 7A1
CYP8B1: Cytochrome P450 8B1
DCA: Deoxycholic acid
FGF: Fibroblast growth factor
FGF15: Fibroblast growth factor 15
FGF19: Fibroblast growth factor 19
FGFR4: Fibroblast growth factor receptor 4
FXR: Farnesoid X receptor
GGT: Gamma-glutamyltransferase
HSC: Hepatic stellate cell
IBABP: Intestinal bile acid binding protein
iNOS: Inducible nitric oxide synthase
KO: Knockout
LCA: Lithocholic acid
LPS: Lipopolysaccharide
LT: Liver transplantation
MCA: Muricholic acid
MRP2: Multidrug resistance-associated protein 2
NASH: Non-alcoholic steatohepatitis
NR: Nuclear receptor
NTCP: Sodium taurocholate co-transporting polypeptide
OCA: Obeticholic acid
OSTα: Organic solute transporter alpha
OSTβ: Organic solute transporter beta
PBC: Primary biliary cholangitis
PFIC2: Progressive familial intrahepatic cholestasis type 2
PSC: Primary sclerosing cholangitis
PXR: Pregnane X receptor
SHP-1: Small heterodimer partner 1
UDCA: Ursodeoxycholic acid
WT: Wild type.
